# Efficacy analysis of C5V chemotherapy combined with transcatheter subcutaneous radiofrequency ablation in the treatment of children with advanced (Stage III/IV) hepatoblastoma

**DOI:** 10.12669/pjms.38.7.5899

**Published:** 2022

**Authors:** Yan-wei Qi, Wei-dong Liu, Lei Gao, Ying-xin Gong

**Affiliations:** 1 Yan-wei Qi, Second Department of General Surgery, Hebei Children’s Hospital, Shijiazhuang 050031, Hebei, China; 2 Wei-dong Liu, Second Department of General Surgery, Hebei Children’s Hospital, Shijiazhuang 050031, Hebei, China; 3 Lei Gao, Second Department of General Surgery, Hebei Children’s Hospital, Shijiazhuang 050031, Hebei, China; 4 Ying-xin Gong, Second Department of General Surgery, Hebei Children’s Hospital, Shijiazhuang 050031, Hebei, China

**Keywords:** C5V regimen, Transductal subcutaneous radiofrequency ablation, Advanced hepatoblastoma, Treatment

## Abstract

**Objectives:**

To evaluate the clinical efficacy of C5V chemotherapy combined with transcatheter subcutaneous radiofrequency ablation in the treatment of children with advanced (stage III/IV) hepatoblastoma.

**Methods:**

Eighty children with advanced (Stage III/IV) hepatoblastoma were admitted in Hebei Children’s Hospital from May 2019 to September 2021 randomly divided into two groups: control group and experimental group, with 40 cases in each group. Children in the control group received C5V chemotherapy, while those in the experimental group received C5V chemotherapy combined with transcatheter subcutaneous radiofrequency ablation. After treatment, the treatment effect, adverse drug reactions, AFP, ALT, AST, HBG and other indicators of the two groups were compared and analyzed. And the difference in survival rate and recurrence rate between the two groups was compared and analyzed.

**Results:**

The total efficacy of the experimental group was 67.5%, which was significantly better than 45% of the control group (p=0.04). The incidence of adverse drug reactions in the experimental group was 50%, while that in the control group was 35% (p=0.15). After treatment, AFP, ALT and AST in the experimental group were significantly lower than those in the control group, while the HBG was slightly higher than that of the control group (p=0.03). Moreover, the overall survival rate of the experimental group was significantly higher than that of the control group, and the recurrence rate was significantly lower than that of the control group.

**Conclusion:**

C5V chemotherapy combined with transcathetal subcutaneous radio fascial ablation is a safe and effective regimen for children with advanced (stage III/IV) hepatoblastoma, boasting definite efficacy and no increase in adverse reactions.

## INTRODUCTION

Hepatoblastoma (HB) is the most common malignant liver tumor in children, accounting for about 80% of pediatric liver tumors.^1^ HB usually lacks specific clinical manifestations in the early stage. Radical resection is the gold standard for the treatment of HB^2^, but usually, only approximately 30% of children have the chance of radical resection at the time of diagnosis because liver space-occupying is usually large in volume, the lesion range involves multiple lobes of the liver, and important structures such as the hepatic hilum cannot be preserved during the surgery, or enough normal liver tissue cannot be preserved due to the large range of resection.^3^ Consequently, more than half of the children have lost the best surgical opportunity when they see a doctor, especially for children with advanced (stage III/IV) HB. Despite neoadjuvant chemotherapy can significantly downgrade and increase the chance of tumor resection, the effect is relatively limited, and the risks of surgery and postoperative infection are more obvious.^4^ The combined treatment of multiple regimens has certain advantages for children with advanced HB.^5^ It is considered in related studies^6^ that cisplatin-containing chemotherapy was an effective treatment for standard hepatoblastoma in children, and radiofrequency ablation is a safe and feasible method for local treatment of HB in children.^7^ In this study, C5V chemotherapy combined with transcathetal subcutaneous radiofrequency ablation was utilized to treat children with advanced (stage III/IV) hepatoblastoma, and certain effects were achieved.

## METHODS

Eighty children with advanced (stage III/IV) hepatoblastoma were admitted in Hebei Children’s Hospital from May 2019 to September 2021 randomly divided into two groups, with 40 cases in each group. No significant difference can be seen in the comparison of general data between the two groups, which was comparable between the two groups (Table-I).

**Table I:** Comparative analysis of general data between the experimental group and the control group (*X̅*±*S*) n=40.

Indicators	Experimental group	Control group	t/χ^2^	p
Age (years old)	4.25±2.01	4.32±1.89	0.30	0.77
Male (%)	22 (55%)	24 (60%)	0.20	0.65
***Pathological types***				
Simple fetal type (%)	25 (62.5%)	22 (55%)	0.46	0.50
Embryonic type (%)	5 (12.5%)	8 (20%)	0.83	0.36
Mixed type (%)	6 (15%)	7 (17.5%)	0.09	0.76
Others (%)	4 (10%)	3 (7.5%)	0.16	0.70
***Clinical staging>***				
III (%)	27 (67.5%)	24 (60%)	0.49	0.48
IV (%)	13 (32.5%)	16 (40%)	
AFP (ng/L)	44673.46±3132.75	46755.71±3127.63	1.36	0.38
ALT (U/L)	197.66±27.43	197.37±25.93	0.05	0.96
AST (U/L)	213.38±36.23	214.08±35.57	0.09	0.93

p>0.05.

### Inclusion criteria:

- Children aged ≤14 years;
- Children with locally advanced (stage III/IV) hepatoblastoma^8^;
- Children with lesions can be accurately assessed by CT, MRI and other imaging methods;
- Children whose pathological results of liver tumor puncture is hepatoblastoma;
- Children with complete clinical data and whose family members agree and cooperate with this study and sign an informed agreement;
- Children with no contraindications with drugs used in this study.

### Exclusion Criteria:

- Children with malignant tumors at other sites;
- Children who have recently taken relevant drugs that affect this study, such as other immunosuppressants and hormones;
- Children with extrahepatic metastasis.

### Ethical Approval

The study was approved by the Institutional Ethics Committee of Hebei Children’s Hospital on April 01, 2019(No.:20190791), and written informed consent was obtained from all participants.

The control group only received the C5V chemotherapy regimen: cisplatin + 5-fluorouracil + vincristine: cisplatin 90 mg/m2, continuous intravenous drip ≥6 h in darkness, day one; 5-fluuracil 600 mg/m2, intravenous for 4 h, day 2; vincristine 1.5 mg/m2 intravenous bolus injection (single maximum dose ≤2 mg), day two. One chemotherapy cycle is given every 21 days, with a total course of treatment of 4-6 cycles. Alpha-fetoprotein (AFP) was reviewed every chemotherapy cycle, and abdominal ultrasound, CT and other imaging examinations were reviewed every two cycles to evaluate the effect of chemotherapy.

The experimental group underwent radiofrequency ablation after two courses of chemotherapy on the basis of the control group. The surgery was performed under general anesthesia, supine or left decubial position, and a multi-section ultrasound probe was scanned from the right intercostal space, under the costal margin and under the xiphoid process. The ablation target area and ablation depth were planned, and the treatment parameters were adjusted to maintain the treatment frequency of 0.8-1 MHz. Ablation was performed inwards from 1 cm outside the tumor tissue, repeated ablation was performed on the deep area of the tumor tissue. The treatment was performed once at an interval of one to two days, for a total of two to six times.

### Observation Indicators: Efficacy evaluation:

All children are evaluated for efficacy after every two treatment cycles. The tumor was evaluated according to Response Evaluation Criteria in Solid Tumors 1.0 (RECIST1.0)^9^: complete response (CR): complete disappearance of the lesion; partial response (PR): the sum of the measured diameters of the target lesion decreased by 30% relative to baseline; Stable disease (SD): the maximum diameter of the lesion was reduced by 25%-50%. Total effective rate = number of (CR+PR) cases/total number of cases×100%.

###  Evaluation of adverse drug reactions:

Adverse drug reactions of the two groups after one treatment cycle, including bone marrow transplantation, gastrointestinal reactions, liver and kidney function injury, fever, pain, and so on, were recorded;

Comparative analysis of laboratory examination indicators between the two groups: fasting blood was taken to detect AFP, aminotransferase (ALT, AST) and hemoglobin (HBG) in the two groups, and to compare the differences of the above indicators. Comparative analysis of follow-up results: All children in the two groups were followed up for 24 months, and their survival rates and recurrence rates were compared and analyzed.

### Statistical Analysis

All the data were statistically analyzed by SPSS 20.0 software, and the measurement data were expressed as (*X̅*±*S*). Two independent sample t-test was used for inter-group data analysis, paired t test was used for intra-group data analysis, and c^2^ was adopted for rate comparison. P<0.05 indicates a statistically significant difference.

## RESULTS

The total effective rate of the experimental group after treatment was 67.5%, which was significantly superior to 45% of the control group (p=0.04) (Table-II).The comparative analysis of the incidence of adverse drug reactions between the two groups after treatment showed that the incidence of adverse reactions in the experimental group was 50%, which was higher than that in the control group (40%), with no statistical significance (p=0.15) (Table-III).

**Table II:** Comparative analysis of the efficacy of the two groups (*X̅*±*S*) n=40

Group	CR	PR	SD	PD	Total effective rate
Experimental group	6	21	11	2	27 (67.5%)
Control group	5	13	15	7	18 (45%)
c^2^					4.11
p					0.04

p<0.05.

**Table III:** Comparative analysis of adverse reactions between the two groups after treatment (*X̅*±*S*) n=40.

Group	Bone marrow suppression	Gastrointestinal reaction	Abnormal liver and kidney function	Fever	Pain	Incidence
Experimental group	7	2	4	3	4	20 (50%)
Control group	7	1	5	0	1	14 (35%)
c^2^						2.05
p						0.15

p<0.05.

**Fig.1:**
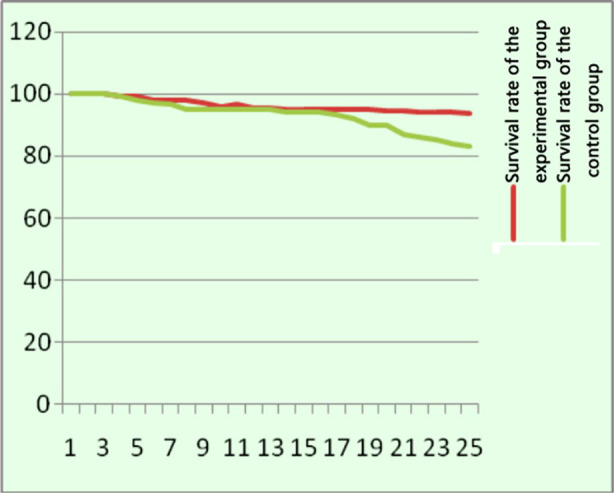
Comparative analysis of the survival rate of the two groups (n=40).

**Fig.2:**
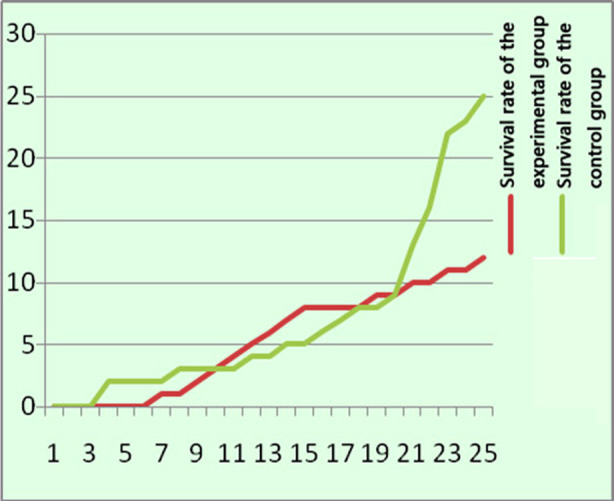
Comparative analysis of the recurrence rate of the two groups (n=40).

After treatment, the AFP, ALT, AST and HBG levels were decreased in both groups, with a statistically significant difference compared with before treatment (p<0.05). Moreover, AFP, ALT and AST in the experimental group were significantly lower than those in the control group after treatment, while the HBG level was slightly higher than that of the control group (Table-IV).The follow-up results showed that the survival rate of the experimental group was 93.5%, and that of the control group was 83.6%, with a statistically significant difference (c2=4.73, p=0.03) (Fig.1). The recurrence rate was 12% in the experimental group and 25% in the control group, with a statistically significant difference (c2=5.60, p=0.02) (Fig.2).

**Table IV:** Comparative analysis of laboratory examination indicators between the two groups before and after treatment (*X̅*±*S*) n=40.

Indicators	Observation points	Experimental group	Control group	t	p
AFP (ng/L)	Before treatment	44673.46±3132.75	46755.71±3127.63	1.36	0.38
	After treatment*	6874.46±465.83	6942.81±545.83	3.56	0.00
AST (U/L)	Before treatment	213.38±36.23	214.08±35.57	0.09	0.93
	After treatment*	83.45±25.30	126.37±26.07	7.47	0.00
ALT (U/L)	Before treatment	197.66±27.43	197.37±25.93	0.05	0.96
	After treatment*	93.64±27.02	114.35±30.86	3.19	0.00
HBG (g/L)	Before treatment	113.27±30.62	113.40±30.37	0.16	0.87
	After treatment*	103.53±21.49	92.74±23.06	2.16	0.03

* p<0.05.

## DISCUSSION

HB accounts for about 1% of pediatric malignancies and 75% to 80% of primary liver malignancies.^10^,^11^ Surgery is the gold standard for the treatment of HB. According to the study^12^, more than half of the children undergoing radical resection lived for more than three years. However, due to the lack of specific clinical manifestations in the early stage of liver disease, more than half of children have no chance of radical treatment at the first diagnosis. Children with HB are sensitive to chemotherapy.^13^ Studies have shown that platinum-containing regimens are the mainstay of initial treatment for children with HB.^14^ However, chemotherapy is a palliative treatment for children. It has been shown in the study^15^ that 60% of children with HB still had residual cancer nests after chemotherapy, and the maximum diameter of residual cancer nests was 11.2mm. The multidisciplinary treatment regimen of percutaneous radiofrequency ablation (RFA) combined with chemotherapy also provides safe and effective treatment options for children with HB.^16^

RFA is an image-guided approach to accurately insert single-stage or multipole ablation electrodes into the tumor site. It transmits pulse energy to tumor tissue, causing tumor tissue to coagulate and necrotic. It was confirmed in the study of Liu et al.^17^ that the first ablation rate of radiofrequency ablation was 80%, without serious complications. Chemotherapy combined with radiofrequency ablation can control tumor growth without affecting liver function damage, thus providing favorable conditions for prolonging the survival of children with HB.^18^ Wang et al.^19^ believed that tumor recurrence after RFA for hepatoblastoma is a key factor affecting the prognosis of patients. Chemotherapy, as a potential therapeutic agent for the treatment of tissues with residual HB, has complementary advantages with RFA, and is expected to become a new means of palliative care.^20^ It was suggested in this study that AFP, ALT, AST and other indicators in the experimental group were significantly lower than those in the control group after treatment, with a statistically significant difference (p=0.00). HBG was slightly higher than that of the control group, with a statistically significant difference (p=0.03), which confirms the above point of view.

As confirmed by this study, the total efficacy of C5V chemotherapy combined with transcatheter subcutaneous radiofrequency ablation in the treatment of children with advanced (stage III/IV) HB was 67.5%, and that of the control group was 45%, with a statistically significant difference (p=0.04). The incidence of adverse drug reactions in the experimental group was 50%, while that in the control group was 35%, showing a statistically significant difference (p=0.15). Moreover, the overall survival rate of the experimental group was 93.5%, and that of the control group was 83.6%, with a statistically significant difference (c2=4.73, p=0.03). The recurrence rate was 12% in the experimental group and 25% in the control group, with a statistically significant difference (c2=5.60, p=0.02).

### Limitations of the study

It includes a small number of samples were included with a short follow-up time, and most of the selected cases were older children to ensure the safety of the study. In view of this, in future clinical work, the sample size will be further expanded, follow-up time will be extended, so that the study content will be more complete and more children will benefit from it.

## CONCLUSIONS

C5V chemotherapy combined with transcathetal subcutaneous radiofascial ablation is a safe and effective regimen for children with advanced (stage III/IV) hepatoblastoma, boasting definite efficacy and no increase in adverse reactions.

### Authors’ Contributions:

**YWQ** & **YXG:** Designed this study,prepared this manuscript, are responsible and accountable for the accuracy and integrity of the work.

**WDL:** Collected and analyzed clinical data.

**LG**: Data analysis, significantly revised this manuscript.
